# Utilization Patterns and Costs of Ocular Amniotic Membrane Grafts in the Medicare Population

**DOI:** 10.1016/j.ophtha.2025.08.023

**Published:** 2025-08-26

**Authors:** Daniel G. Vail, Eric Nudleman, Luay Abdeljaber, Md Enamul Haque, Suzann Pershing

**Affiliations:** 1 Viterbi Family Department of Ophthalmology, Shiley Eye Institute, University of California, San Diego, La Jolla, California; 2 Byers Eye Institute, Department of Ophthalmology, Stanford University School of Medicine, Palo Alto, California; 3 VA Palo Alto Health Care System, Palo Alto, California

**Keywords:** Amniotic membrane graft, CMS spending, Dry eye, Health care spending, Medicare, Reimbursement policy, Skin substitutes

## Abstract

**Purpose:**

Recent investigations from the Office of the Inspector General and *The New York Times* have raised concerns about the costs to Medicare of skin substitutes, documenting pervasive fraudulent billing practices outside of ophthalmology. These investigations have not covered the use of tissue in eye care, despite similar reimbursement incentives for the use of sutureless ocular amniotic membrane grafts (AMGs). We examine the volume, cost, and clinical indications associated with sutureless AMGs in eye care.

**Design:**

Retrospective cohort study of fee-for-service Medicare patients who received sutureless AMGs and eye care providers who bill for sutureless AMGs.

**Participants:**

Nationally representative 20% sample of Medicare Part B claims for sutureless AMGs between 2011 and 2020.

**Methods:**

We use longitudinal patient-level and provider-level data to specify regression models for our primary outcomes. We estimate Cox proportional hazards survival models for time-to-event analyses and logistic regression models for binary outcomes.

**Main Outcome Measures:**

Clinical indications for AMG use, time from patients’ first encounter with a provider to their first AMG, use of AMGs for dry eye, and costs to Medicare.

**Results:**

Dry eye accounted for 44% of all sutureless AMG use in 2020, increased from 7% in 2011. Charges for ocular sutureless AMGs increased from $3.5 million in 2011 to $95.6 million in 2020; charges for AMGs used to treat dry eye increased from $250 000 to $41.4 million over this period. Some 28% of all claims for AMGS were submitted by 1% of providers. Patients are more likely to receive an AMG when seen by an optometrist (hazard ratio, 1.16; *P* < 0.001). The annual costs to Medicare for a patient with dry eye have increased dramatically among providers who use AMGs.

**Conclusions:**

The costs to Medicare for skin substitutes are inflated by reimbursement models that encourage their use even when there is little clinical indication. These concerns may be applicable to eye care as well, where spending on amniotic membrane grafts has increased dramatically, particularly for indications where clinical benefit may be limited. By requiring AMG manufacturers to report accurate sales prices, policymakers could reduce costs without limiting coverage of AMGs for appropriate clinical indications.

**Financial Disclosure(s):**

Proprietary or commercial disclosure may be found after the references.

A recent *New York Times* investigation concluded that substantial fraudulent billing for wound care occurs in fee-for-service Medicare.^1^ The investigation focused on the use of “skin substitutes” used for treatment of patients with chronic wounds and described a confluence of factors that have contributed to overuse of preserved tissue bandages in wound care, including a “buy and bill” reimbursement design combined with readily available manufacturer discounts, which allow administering physicians to purchase tissue for much less than they are reimbursed for it by the Centers for Medicare & Medicaid Services (CMS). The authors report that billing for skin substitutes in Medicare has increased rapidly, reaching $10 billion in 2024, and that the increase has little clinical justification. Similar concerns about overuse of skin substitutes were documented by the Office of the Inspector General in 2023.^2^

The use of amniotic membrane grafts (AMGs) in ophthalmology has not received similar attention, despite relying on a reimbursement model similar to the use of skin substitutes in wound care. Ophthalmologists use preserved tissue, specifically AMGs, for treating a host of clinical indications, including corneal ulcers,^3^ nonhealing epithelial defects,^4^ trauma, and inflammation (including burns),^5^ and as a conjunctival substitute during pterygium excision, glaucoma surgery, oculoplastic surgery, and ocular surface reconstruction (e.g., in the setting of ocular malignancy or trauma).^6^ Amniotic membrane grafts have been used in ophthalmology since the 1940s, and their use increased in the 1990s with the advent of novel methods for preserving and storing the tissue.^6^ In 2003, the US Food and Drug Administration approved ProKera, an ophthalmic conformer with an incorporated amniotic membrane that can be applied to the ocular surface without sutures or tissue glue. Of note, although the stated indication for ProKera is for ocular surface disease and the purported benefits of the device are focused on the amniotic membrane that it contains, the device received 510(k) clearance because it was deemed to be substantially similar to a symblepharon ring, which provides mechanical support and helps prevent ocular surface damage from scarring, but lacks an amniotic membrane.^7^ Competing sutureless AMG devices have subsequently been introduced.

Sutureless AMGs are easier for clinicians to apply in the clinic compared with AMGs that require sutures or tissue glue to maintain their position. Additionally, they can be applied by optometrists and by other clinicians who are not credentialed to perform ophthalmic surgery. Amniotic membrane grafts have proven clinical benefits in certain severe vision-threatening conditions such as Stevens-Johnson Syndrome and severe chemical burns;^8,9^ however, the clinical benefits of sutureless AMGs in some other indications are controversial. For example, some studies have identified modest potential benefits associated with AMG use in certain cases of bacterial keratitis^10^ and neurotrophic corneal ulcers,^11^ whereas others have found no clinically significant benefit in the setting of recurrent pterygia^12^ and some chemical injuries.^13^ The use of AMGs in management of dry eye disease is an area of ongoing research and controversy. Dry eye disease is prevalent and multifactorial, with protean management strategies, and there is evidence that some patients with severe or refractory dry eye may benefit from the use of AMGs to treat confluent epithelial defects or to reduce ocular surface inflammation either as a primary treatment or as an adjuvant to other therapies.^14,15^ A randomized control trial found that patients with moderate-to-severe dry eye who received AMG under a bandaged contact lens had improvement in symptoms and signs of dry eye disease compared with patients who only received a bandaged contact lens.^16^

The financial benefits of AMGs for providers are more clear-cut than the clinical benefits for patients. A cursory review of online resources for eye practitioners demonstrates many instances of authors touting the financial awards associated with using AMGs.^17–19^ Some of these resources advertise discounts on purchasing AMGs from manufacturers or emphasize the differences between the tissue acquisition costs and the prices paid for tissue reimbursement; a 2022 article in the *Review of Optometric Business* describes a case study practice that typically acquires each ProKera for $700 and receives $1300 in reimbursement for placing the AMG.^18^ This $600 profit margin exceeds the physician compensation for routine cataract surgery (Common Procedural Terminology [CPT] code 66984) in the Medicare Physician Fee Schedule for 2022.^20^ The discrepancy between the reimbursement and the cost of tissue acquisition arises because CMS reimburses providers based on the list price of the tissue (the Wholesale Acquisition Cost) rather than the true cost of the tissue after taking into account rebates and manufacturer discounts (the average sales price [ASP]). In 2021, Congress attempted to address this by requiring manufacturers of skin substitutes and other Part B products to report their true ASP to CMS (after accounting for all manufacturer discounts), but a 2023 report from the Office of the Inspector General noted that approximately half of manufacturers had failed to comply with the new requirements.^2^ The report concluded that transitioning to a system in which products were reimbursed at their ASP would save CMS more than $330 million each year; these estimates are restricted to skin substitutes for wound care and do not include any ophthalmic costs.

Although billing for skin substitutes in the Medicare population is a topical concern for policymakers, the use of ophthalmic AMGs in fee-for-service Medicare has not been previously analyzed. We use a 20% sample of Medicare Part B fee-for-service claims to study the use of AMGs within ophthalmology from 2011 (the year that the CPT code for sutureless AMG was introduced) to 2020 (the most recent year for which claims-level Part B data are available). We focus on the use of sutureless ocular AMGs and use detailed patient-level and provider-level data to characterize the volume of AMGs billed to Medicare, the clinical indications for their use, relative use by optometrists and ophthalmologists of different subspecialties, and billing patterns that may be consistent with inappropriate use of ocular AMGs. We further investigate how each of these patterns has changed over time and whether use of ocular AMGs is associated with higher-cost management of eye care patients in general. Finally, we discuss the implications that our analysis has for policymakers and practicing ophthalmologists.

## Methods

We use a 20% sample of fee-for-service Medicare Part B claims from 2011 to 2020 for this analysis.^21^ This is a nationally representative sample of the population insured under traditional fee-for-service Medicare and includes patient-level diagnostic and procedural information. The sample is selected at the patient level rather than the claim level, meaning that all claims are available for the 20% of patients included in the sample (as opposed to being a random sample of all medical claims that exist across the entire Medicare population). Patients are followed longitudinally over time with unique identifiers,^22^ so that records can be consolidated even in instances where patients disenroll and re-enroll in Medicare. Our data include physician identifiers, which allow us to characterize physician practice patterns. As a secondary analysis of de-identified data, our investigation was reviewed by the Stanford University Institutional Review Board and deemed to be minimal risk (Institutional Review Board eProtocol #41401). We adhere to the tenets of the Declaration of Helsinki and to the Strengthening the Reporting of Observational Studies in Epidemiology guidelines in reporting our results. The requirement for informed consent was waived because this was a retrospective analysis of administrative claims data and is not human subjects research.

### Sample Selection: Patient-level Variables

The billing code for “placement of amniotic membrane on the ocular surface, without sutures” (CPT 65778) was approved for use in 2011. We identify all instances of this CPT code in Part B claims from 2011 to 2020 (the last year for which we have CMS data available). We use these claims to select all patients who received a sutureless ocular AMG during the study period. For each patient, we identify the first instance that they received a sutureless ocular AMG and the National Provider Identifier (NPI) of the clinician who billed for the AMG. Additionally, we identify the diagnosis code associated with the AMG procedure.

These data were used to establish each patient’s first visit with the provider who administered their AMG and the first time they were diagnosed with the condition used to justify placement of the AMG, allowing us to calculate the time between a patient’s first visit and their first ocular AMG. As such, we retrieve all instances before and after placement of the AMG when the patient met any eye care provider (provider identification and definition of provider type are described in detail below). Finally, we use the complete medical claims for each patient to establish their ophthalmic and nonophthalmic medical history preceding receipt of their AMG. For example, we construct variables for any history of dry eye, corneal ulcer (infectious, noninfectious, and neurotrophic), recurrent corneal erosions, corneal abrasions, keratitis and conjunctivitis, corneal edema, and eyelid abnormalities. We create additional demographic variables including patient sex and age, and construct a measure of patients’ systemic health at the time of their first AMG visit using the Elixhauser comorbidity index, a validated vector of systemic diagnoses frequently used in similar studies.^23^

We restrict our analysis to patients with at least 365 days of continuous enrollment in Medicare during the study period, with a look-back period of 180 days preceding their first receipt of an ocular AMG. All diagnoses were specified using *International Classification of Diseases*, 9^th^ and 10^th^ Editions diagnosis codes, and procedures were identified using Common Procedural Terminology (CPT) codes. A complete list of variable definitions and associated diagnostic and procedural codes is available in Table S1 (available at www.aaojournal.org).

### Sample Selection: Provider-level Variables

We specify a number of variables calculated at the provider level to characterize variation in the use of AMGs by different practitioners and to estimate patients’ probability of receiving an AMG based on which provider they visit. We select all ophthalmologist and optometrist NPIs using a 2-stage identification process. Medicare claims include a native categorical variable to identify provider type. However, this variable is based on provider self-identification at the time of their initial enrollment in the database and therefore contains inaccuracies (e.g., a provider who initially trained in internal medicine before completing a residency in ophthalmology might be categorized as an internal medicine physician). We select all self-identified optometry and ophthalmology NPIs, restrict our sample to practitioners with at least 365 days of continuous enrollment in the Medicare database, and then further categorize ophthalmologists by their billing patterns, using an algorithm described in other analyses of commercial claims data.^24^ For example, we define anterior segment surgeons as practitioners who bill, on average, at least 50 specialized anterior segment CPT codes each year that they are enrolled in the database (in this 20% sample of Part B claims, this would equate to providers billing for at least 250 anterior-segment CPT codes each year solely among their fee-for-service Medicare patients, and would not include codes billed for Medicare Advantage or non-Medicare patients). Similarly, we define glaucoma surgeons as those billing at least 30 specialized glaucoma CPT codes each year and retina surgeons as those billing at least 50 specialized vitreoretinal CPT codes annually. A complete list of provider-level variable definitions is available in Table S2 (available at www.aaojournal.org).

After identifying optometrists and ophthalmologists in our sample, we characterize their practice patterns by analyzing their submitted claims for all patients in the Part B database (not just those patients who received AMGs).

### Analysis

We characterize billing for sutureless AMG from 2011 to 2020, stratified by provider type (optometry, ophthalmology, and ophthalmic subspecialty). We characterize the most common diagnoses associated with AMG claims over time and characterize changes in billing patterns stratified by provider type. Furthermore, we estimate costs over time associated with use of AMGs, stratified by diagnosis type and provider category.

We calculate the total number of eye care visits each provider bills each year using both eye-specific codes and general Evaluation and Management codes, the complexity level of those visits (1—5), and the primary diagnosis at each visit. We calculate the unique number of patients seen by each provider each year, the percentage of each provider’s patient population that is composed of patients who have *ever* been diagnosed with dry eye, and the percentage of visits for which the primary diagnosis code associated with AMG placement is dry eye. We calculate the percentage of unique dry eye patients seen by each provider who ever receive an AMG from that provider and the percentage of all dry eye visits for each provider that are associated with a billing code for AMG. Finally, we calculate the costs to CMS of each visit for each provider, as well as the average cost of each unique dry eye patient for each provider each year.

We calculate patients’ time from their first encounter with the provider who bills for their AMG, and for a subsample of patients who received AMG for dry eye, we calculate the time from their first diagnosis with dry eye to their first AMG. We specify Cox proportional hazards survival models to estimate patients’ probability of receiving an AMG after accounting for their primary diagnosis, the category of eye provider who diagnoses them, patient age, sex, and systemic comorbidities. We estimate patients’ probabilities of receiving multiple AMGs using logistic regression models that account for patients’ diagnostic history and provider-level characteristics.

## Results

The 20% Medicare Part B sample includes 49 180 instances of billing codes for sutureless ocular AMG between 2011 and 2020, comprising 23 894 unique patients who met inclusion criteria for this study. The mean age of patients at the time of their first AMG was 74.6 years. The majority (68.0%) of the sample was female. Claims for placement of a sutureless AMG were submitted by 6416 unique providers during the study period. The distribution of claims was heavily right-skewed; 1% (N = 64) of eye providers submitted 28.2% of all claims for AMGs during the study period.

**Figure 1.**
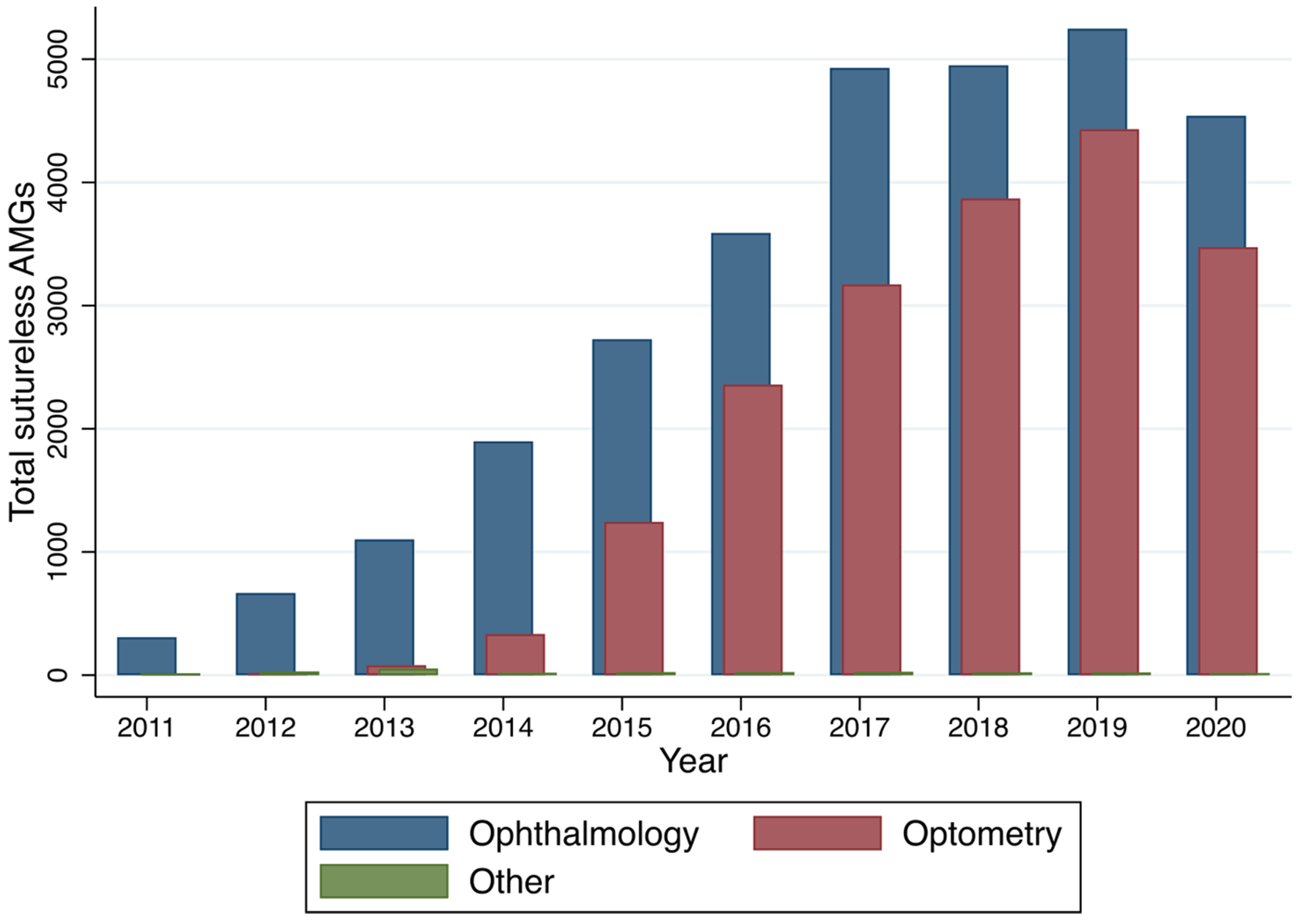
Total claims for sutureless amniotic membrane grafts (AMGs) submitted by optometrists and ophthalmologists in a 20% sample of Medicare Part B, 2011—2020.

Frequency of sutureless AMGs increased from 319 in 2011 to 8031 in 2020 in this sample. Increased use of AMGs in eye clinics has been accompanied by a change in the indications for which they are used. In 2011, when clinicians first began using the CPT code for sutureless AMGs, diagnosis codes for corneal ulcers and corneal epithelial defects (including corneal abrasions and recurrent corneal erosions) accounted for 39.8% and 12.2%, respectively, of all sutureless AMG claims. Sutureless AMGs were only billed for management of dry eye in 7.2% of cases. By 2020, dry eye accounted for 44.2% of all sutureless AMG diagnosis claims (Fig 1).

**Figure 2.**
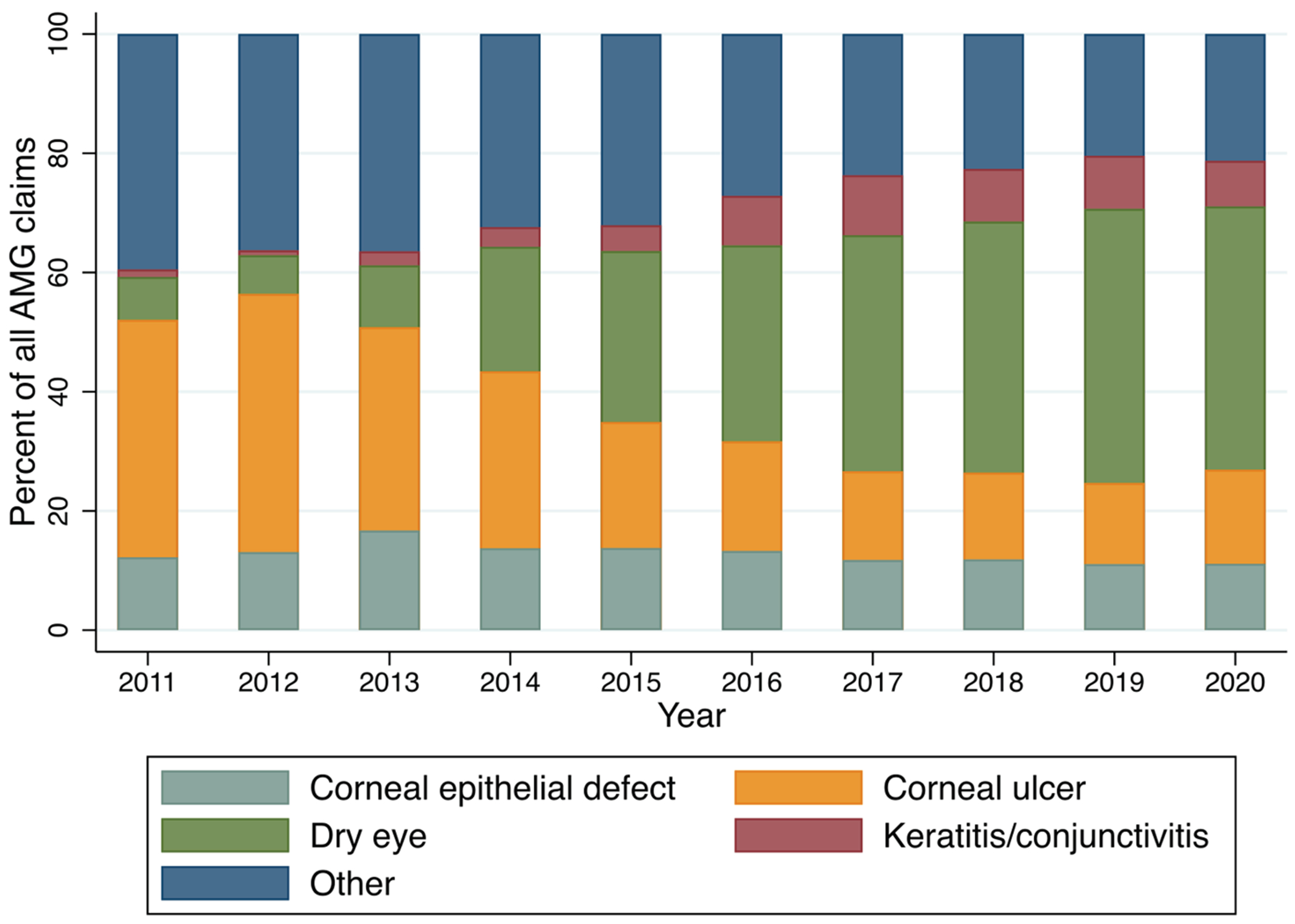
Primary diagnosis code associated with claims for sutureless amniotic membrane grafts (AMGs) in a 20% sample of Medicare Part B, 2011—2020.

Use of AMGs increased dramatically among both ophthalmologists and optometrists. Uptake among optometrists started later but increased rapidly; optometrists accounted for 0% of submitted claims in 2011 compared with 43.3% of claims in 2020 (Fig 2). AMG use among ophthalmologists occurs primarily among anterior segment specialists and comprehensive ophthalmologists (Fig S3, available at www.aaojournal.org). Dry eye diagnosis codes are particularly predominant in claims submitted by optometrists compared with claims submitted by ophthalmologists (Fig S4, available at www.aaojournal.org). Dry eye codes account for the majority (59%) of diagnoses associated with AMG placement by optometrists in 2020, whereas other diagnoses (including corneal ulcer, epithelial defect, and keratitis/conjunctivitis) collectively eclipse dry eye indications among ophthalmologists.

**Figure 5.**
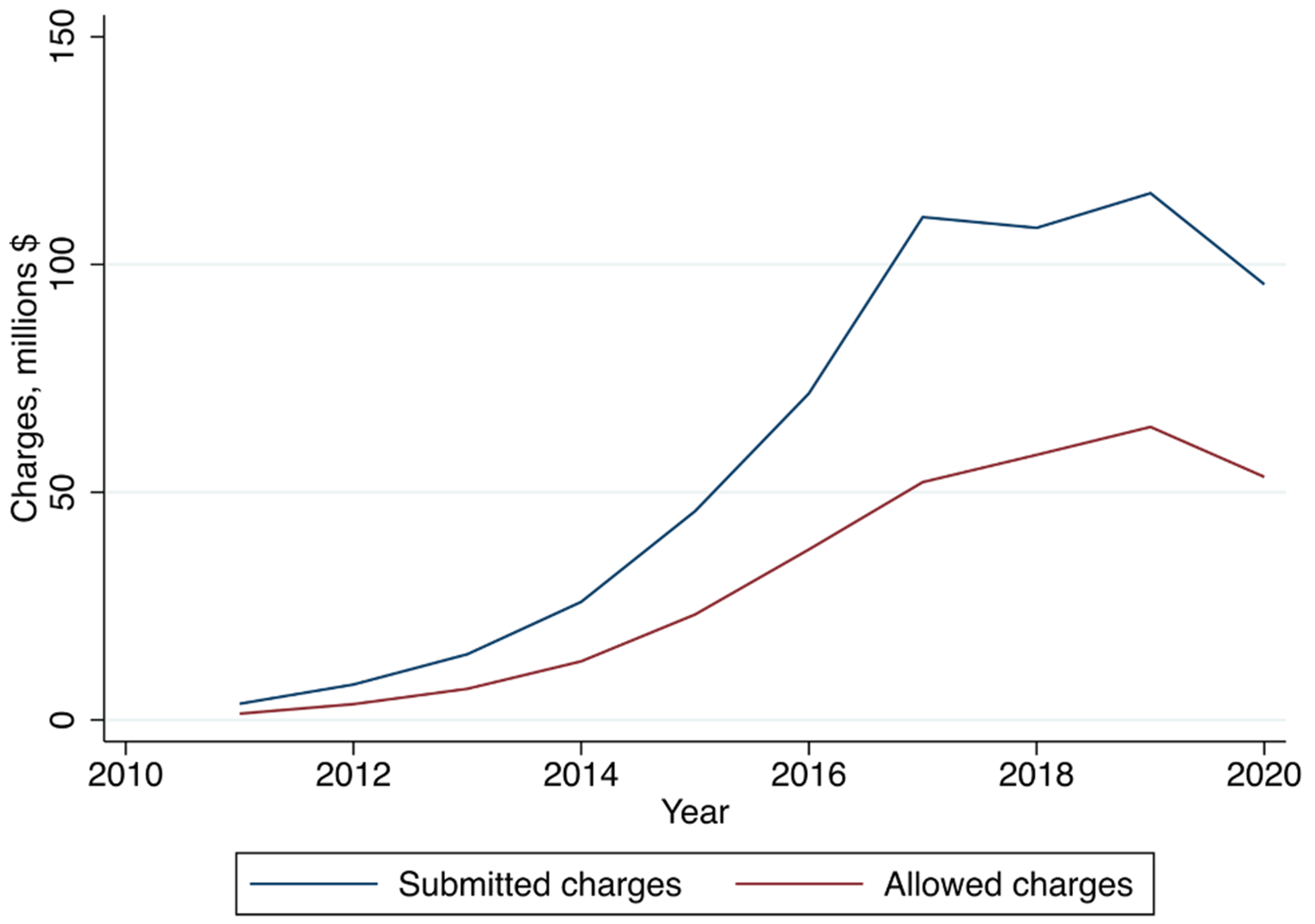
Estimated charges for sutureless amniotic membrane grafts (AMGs) in Medicare Part B, 2011—2020.

**Figure 6.**
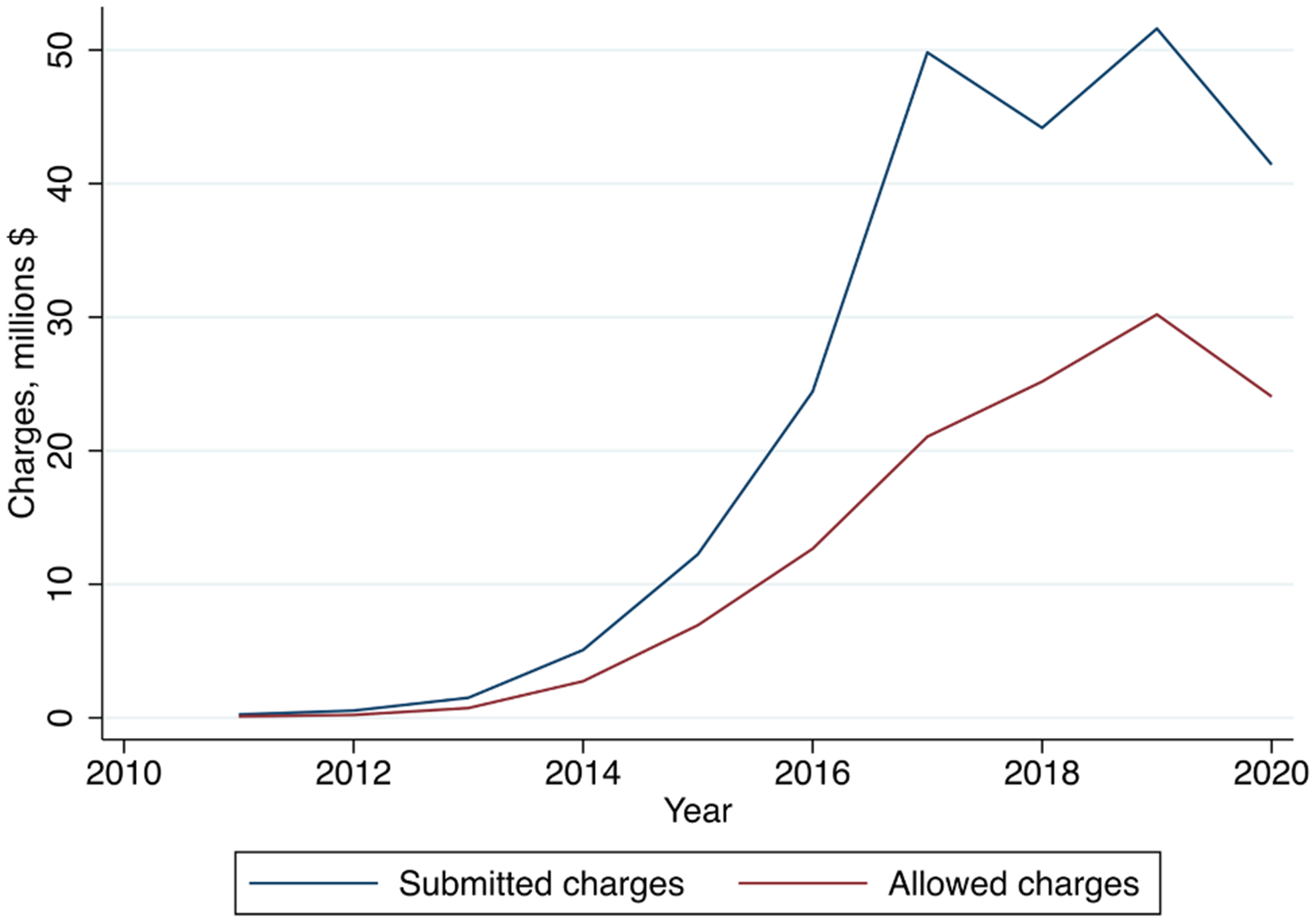
Estimated charges for sutureless amniotic membrane grafts (AMGs) used to treat dry eye in Medicare Part B, 2011—2020.

Extrapolating the results of our 20% sample to the full Medicare fee-for-service population, we estimate the total cost of claims submitted to fee-for-service Medicare for sutureless AMGs increased from $3.6 million in 2011 to $95.6 million in 2020; allowed charges for submitted claims increased during this period from $1.4 million to $53.4 million. These increases are notable because they occurred during a period in which an increasing portion of the Medicare population is covered under Medicare Advantage, rather than fee-for-service Medicare; the total costs of AMGs to the Medicare program as a whole are likely substantially higher (Fig 5). Even more dramatically, the costs of sutureless AMGs specifically for management of dry eye increased from $250 000 in 2011 to $41.4 million in 2020 (submitted charges; allowed charges increased from $110 000 to $24.1 million during this same time period) (Fig 6).

Using longitudinal data from patients who received a sutureless AMG, we specify a Cox proportional hazards model of patients’ time from their initial encounter with the provider who places their AMG until their receipt of the AMG. After accounting for patients’ diagnoses, age, sex, and systemic comorbidities, we find that patients are more likely to be prescribed an AMG when seen by an optometrist compared with an ophthalmologist (hazard ratio, 1.16, *P* < 0.001). Patients were significantly more likely to be given an AMG within 1 week of meeting a provider if they were treated by an optometrist (odds ratio [OR], 4.38, *P* = 0.017). In a subanalysis of patients who received AMG for dry eye, we find that although patients’ time from diagnosis to AMG was not significantly lower overall when treated by optometrists, patients were more likely to receive an AMG at the time of their first-ever diagnosis with dry eye when seen by an optometrist (OR, 1.29, *P* < 0.001), were more likely to receive a second AMG when seen by an optometrist (OR, 1.25, *P* < 0.001), and among patients who received a second AMG, time between first and second AMG was shorter when patients were managed by optometrists (hazard ratio, 1.16, *P* < 0.001) (Table 3). Our results are unchanged when we eliminate the Elixhauser comorbidity index as a model covariate (Table S4, available at www.aaojournal.org).

**Figure 7.**
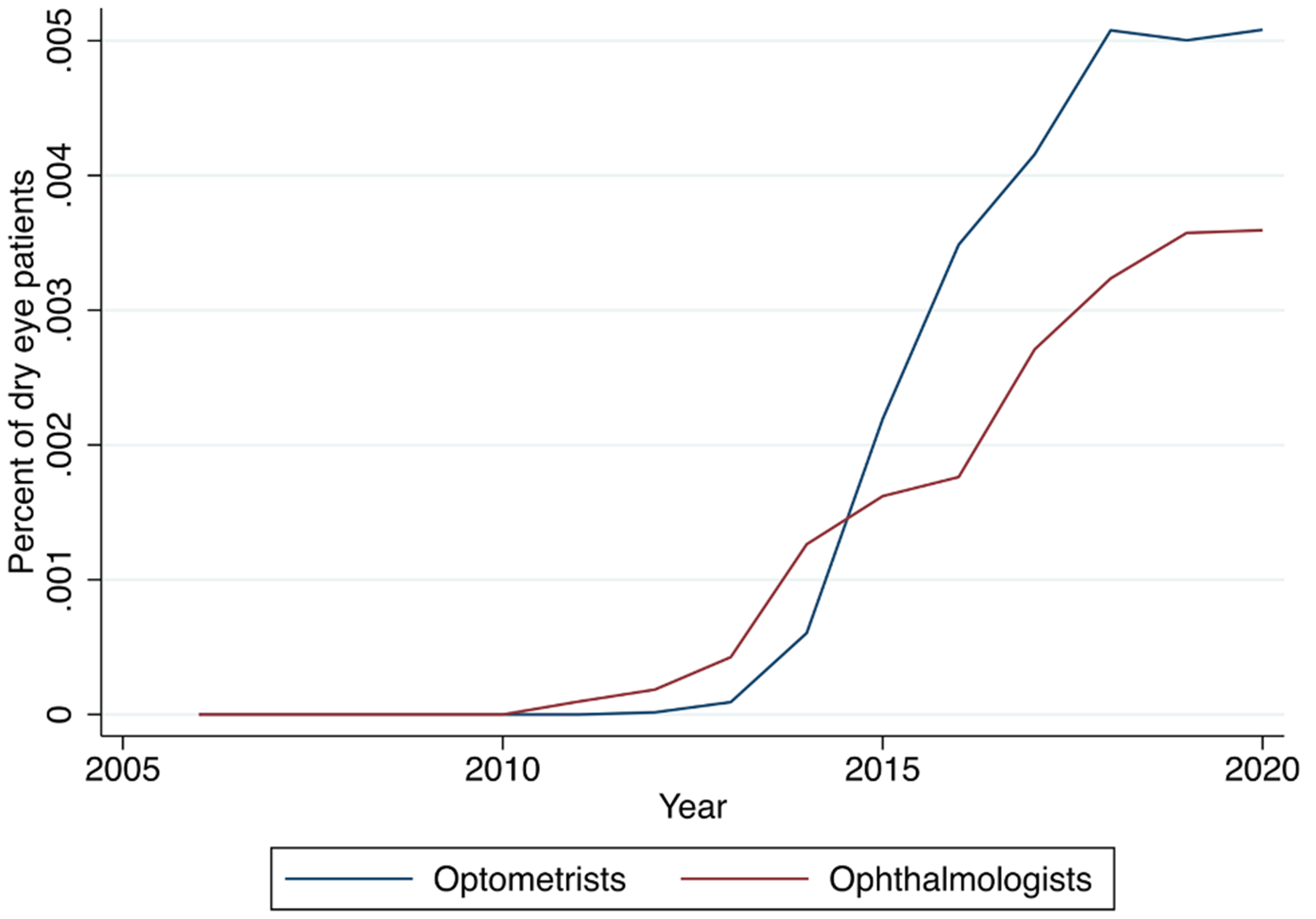
Percentage of dry eye patients who receive a sutureless amniotic membrane graft (AMG) in a 20% sample of Medicare Part B, 2011—2020.

**Figure 9.**
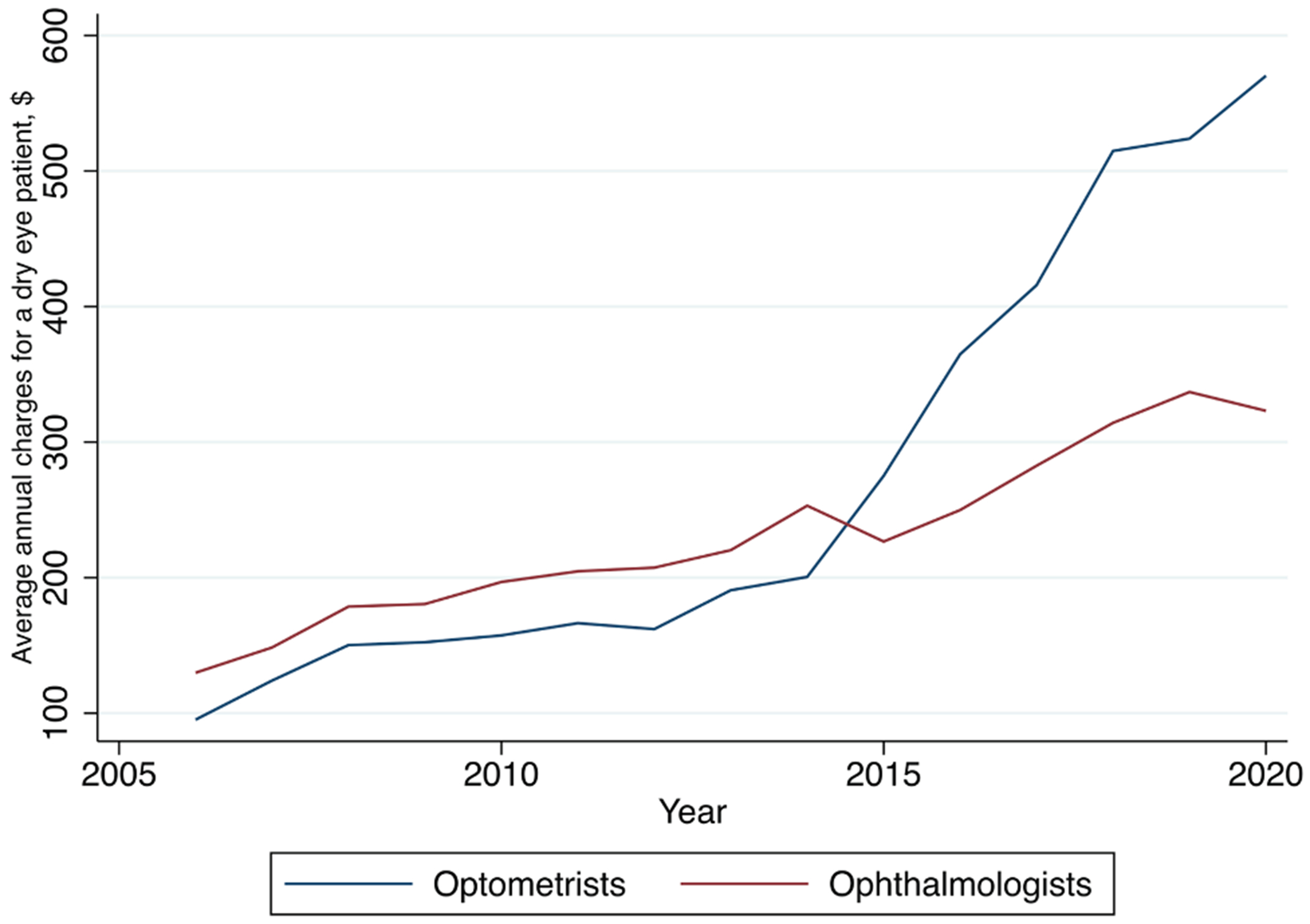
Average annual charges for a dry eye patient among providers who use sutureless amniotic membrane grafts (AMGs) in their practices, in a 20% sample of Medicare Part B, 2011—2020.

In provider-level analyses, we find the percentage of individual providers’ dry eye patients who receive a sutureless AMG has increased over time, on average, and is higher in optometry practices (Fig 7). Although the average annual costs associated with a single dry eye patient were higher among ophthalmology patients than optometry patients (Fig S8, available at www.aaojournal.org), costs for dry eye patients are substantially higher among optometry patients when we restrict the sample to include providers who use at least 1 sutureless AMG per year (Fig 9).

## Discussion

The costs associated with preserved tissue grafts are a topical concern for policymakers, and particularly for CMS. Despite recent investigations from the Office of the Inspector General and *The New York Times* demonstrating pervasive clinically inappropriate use of skin substitutes for wound care, the policy implications of the use of AMGs in eye care have not previously been investigated. We find substantial and increasing costs in the fee-for-service Medicare program associated with the use of sutureless ocular AMGs, and demonstrate that the increased costs that CMS has paid for ocular AMGs from 2011 to 2020 are largely driven by clinicians using AMGs for dry eye.

Use of AMGs is heavily right-skewed, with a small percentage of optometrists and ophthalmologists accounting for a disproportionate share of AMG claims. Patients treated by optometrists were more likely to receive an AMG at their initial visit with the administering provider, more likely to receive an AMG at the time of their visit-ever diagnosis with dry eye, and more likely to receive multiple AMGs. Costs of managing the average dry eye patient have increased over time, particularly among providers who use AMG, and most particularly among optometrists who use AMGs.

Increasing use of ocular AMGs, combined with a reimbursement model similar to the one associated with fraudulent billing for tissue in non-ophthalmic settings, raises concern that financial incentives have contributed to inappropriate use of an expensive technology with limited clinical benefit. Indeed, some cases of ocular AMG use have been directly linked to fraudulent billing.^25^ These outlier cases represent a small portion of all AMG billing, and certainly the preponderance of AMG use within ophthalmology has clinical justification (even the use of AMG for dry eye has purported benefits, and different clinical experts will have different opinions on the utility of AMG for different patients).^6^ But a scenario where limited or conflicting clinical evidence for a treatment is combined with a very clear financial incentive to use the treatment is inefficient from a policy standpoint.

Manufacturer discounts for AMGs can be substantial, allowing clinicians to profit on the difference between their tissue acquisition costs and the list prices for tissue. The effective reimbursement that clinicians are paid to care for a patient who receives an AMG is not a flat rate for placement of the device, but is instead defined by the lowest price they can purchase an AMG and the highest price that CMS will reimburse them for an AMG; in effect, clinicians are being paid on commission by the AMG supplier. The long-term stability of this reimbursement model is uncertain. The Office of the Inspector General has already recommended transitioning to a system where tissue reimbursement accounts for the true acquisition costs after factoring in manufacturing discounts. Although policymakers have a strong incentive to transition to a different reimbursement model that more closely aligns quality of patient care with costs of care, clinicians may as well if a revised reimbursement model is implemented strategically. In an environment where the Physician Fee Schedule has failed to keep pace with inflation, and where eye care has repeatedly been targeted with fee cuts, it is in clinicians’ best interest that policymakers reduce spending by targeting wasteful and inefficient care, rather than by implementing broad cuts in the fee schedule.

As previously discussed, AMGs do play a crucial role in the management of certain ocular conditions, including vision-threatening cases of chemical injuries and autoimmune inflammation.^8,9^ Additionally, there is ongoing research into the use of sutureless AMGs for management of refractory dry eye disease, with evidence that they can improve symptoms compared with other common treatments when used in appropriate patients.^16^ By more closely aligning tissue reimbursement with the true acquisition costs for sutureless AMGs, policymakers could ensure continued coverage for a medical device with established benefits and encourage ongoing research into its adoption for novel clinical indications, while also limiting the costs associated with inappropriate use of AMGs in situations where they are unlikely to benefit patients.

### Limitations

As with all analyses of insurance claims data, we are unable to observe the patient-level clinical information that informs physician decision-making; it is impossible in this analysis to determine whether any individual claim for an AMG is appropriate or not. Additionally, our dataset offers insight solely into the care that patients receive through Medicare; clinical history that precedes their enrollment in Medicare or is financed outside of Medicare (e.g., visits to providers who do not accept Medicare or are not covered by Medicare, and are paid out-of-pocket by the patient) is not observed. It is possible, for example, that patients with dry eye received a diagnosis or treatment for their condition before enrolling in Medicare and are not truly “newly diagnosed” when we first observe them. We use a look-back period to ameliorate the effects of patients’ pre-enrollment clinical histories on our findings, but as with any analysis of patient-level data, our analyses are drawn from a subset of their full clinical histories.

## Supplementary Material

1

2

3

4

5

6

Supplemental material available at www.aaojournal.org.

## Figures and Tables

**Table 3.** Patient-level Models of Receipt of Amniotic Membrane Grafts

Outcome	Model 1	Model 2	Model 3	Model 4	Model 5	Model 6
	*Time from First Encounter with Provider to First AMG*	*Receipt of AMG at First Visit with Provider*	*Time from First Diagnosis with Dry Eye to First AMG*	*Receipt of AMG at First Diagnosis with Dry Eye*	*Time from First AMG to Sec ond AMG*	*Receipt of Multiple AMGs*
Model Specification	Cox Proportional Hazards	Logistic	Cox Proportional Hazards	Logistic	Cox Proportional Hazards	Logistic
Model N	N = 23 059	N = 21 220	N = 6547	N = 8538	N = 9012	N = 23 066
Mean days to outcome	3171	13	1119	2305	106	10 815
Frequency of outcome						
	HR (*P* value)	OR (*P* value)	HR (*P* value)	OR (*P* value)	HR (*P* value)	OR (*P* value)
Provider specialty						
Ophthalmology (base)						
Optometry	1.160 (*P* < 0.001)	3.932 (*P* = 0.031)	1.005 (*P* = 0.816)	1.290 (*P* < 0.001)	1.159 (*P* < 0.001)	1.251 (*P* < 0.001)
Other	1.343 (*P* = 0.002)		2.000 (*P* = 0.230)	4.484 (*P* = 0.110)	1.578 (*P* = 0.101)	2.157 (*P* < 0.001)
Diagnosis associated with AMG						
Corneal abrasion (base)						
Corneal ulcer	1.114 (*P* < 0.001)	0.651 (*P* = 0.730)			1.101 (*P* = 0.028)	1.323 (*P* < 0.001)
Dry eye	0.907 (*P* < 0.001)	0.904 (*P* = 0.905)			1.482 (*P* < 0.001)	2.431 (*P* < 0.001)
Keratitis or conjunctivitis	0.845 (*P* < 0.001)				1.172 (*P* = 0.001)	1.713 (*P* < 0.001)
Other	1.094 (*P* < 0.001)	1.747 (*P* = 0.510)			1.044 (*P* = 0.287)	1.307 (*P* < 0.001)
Sex						
Female (base)						
Male	0.986 (*P* = 0.310)	1.329 (*P* = 0.620)	1.294 (*P* < 0.001)	1.802 (*P* < 0.001)	1.047 (*P* = 0.048)	0.884 (*P* < 0.001)
Age (yrs)	0.997 (*P* < 0.001)	0.990 (*P* = 0.692)	0.978 (*P* < 0.001)	0.977 (*P* < 0.001)	1.000 (*P* = 0.995)	0.993 (*P* < 0.001)
Elixhauser comorbidity index (continuous)	1.015 (*P* < 0.001)	0.957 (*P* = 0.523)	0.957 (*P* < 0.001)	0.960 (*P* < 0.001)	0.987 (*P* < 0.001)	1.018 (*P* < 0.001)

AMG = amniotic membrane graft; HR = hazard ratio; OR = odds ratio.

## Abbreviations and Acronyms:

AMG: amniotic membrane graft
ASP: average sales price
CMS: Centers for Medicare & Medicaid Services
CPT: Common Procedural Terminology
NPI: National Provider Identifier
