# Supplementary material for: Utilization Patterns and Costs of Ocular Amniotic Membrane Grafts in the Medicare Population
Source: Ophthalmology. Author manuscript; Available in PMC 2026 Jul 21. (PMC13387592; doi:10.1016/j.ophtha.2025.08.023)
Supplement: 2 [file NIHMS2182128-supplement-2.pdf]

Appendix Table 2

Common Procedural Terminology Codes								
**Anterior segment codes**	Posterior segment codes	Glaucoma codes	Eye visit codes	E&M codes	AMG codes	AMG supply codes	Sutureless AMG codes	Specialty definitions:
**65400**	67005	66183	92002	99202	65775	V2790	65778	Glaucoma: Bill at least 30 glaucoma CPT codes per year on average
**65410**	67010	66150	92004	99203	65778			Retina: Bill at least 50 posterior segment CPT codes per year on average
**65420**	67015	66155	92012	99204	65779			Anterior segment: Bill at least 250 anterior segment CPT codes per year on average
**65426**	67025	66160	92014	99205	65780			
**65430**	67027	66165	92015	99212	65426			
**65435**	67028	66170	S0620	99213	66999			
**65436**	67030	66172	S0621	99214	68110			
**65450**	67031	66180		99215	68115			
**65600**	67036	66185			68326			
**65710**	67038	66220			68330			
**65730**	67039	66700			66170			
**65750**	67040	66710			66172			
**65755**	67101	66711			66180			
**65760**	67105	66720			66185			
**65765**	67107	66740			V2790			
**65767**	67108							
**65770**	67110							
**65771**	67112							
**65772**	67115							
**65775**	67120							
**65780**	67121							
**65781**	67141							
**65782**	67145							
**65800**	67208							
**65805**	67210							
**65810**	67218							
**65815**	67220							
**65820**	67221							
**65850**	67225							
**65855**	67227							
**65860**	67228							
**65865**	67250							
**65870**	67255							
**65875**	67299							
**65880**								
**65900**								
**65920**								
**65930**								
**66020**								
**66030**								
**66130**								
**66150**								
**66155**								
**66160**								
**66165**								
**66170**								
**66172**								
**66180**								
**66185**								
**66220**								
**66225**								
**66250**								
**66500**								
**66505**								
**66600**								
**66605**								
**66625**								
**66630**								
**66635**								
**66680**								
**66682**								
**66700**								
**66710**								
**66711**								
**66720**								
**66740**								
**66761**								
**66762**								
**66770**								
**66820**								
**66821**								
**66825**								
**66830**								
**66840**								
**66850**								
**66852**								
**66920**								
**66930**								
**66940**								
**66982**								
**66983**								
**66984**								
**66985**								
**66986**								
**66990**								
**66999**								
