# Supplementary material for: Utilization Patterns and Costs of Ocular Amniotic Membrane Grafts in the Medicare Population
Source: Ophthalmology. Author manuscript; Available in PMC 2026 Jul 21. (PMC13387592; doi:10.1016/j.ophtha.2025.08.023)
Supplement: 3 [file NIHMS2182128-supplement-3.pdf]

Table S4: Re-estimating all Models in Table 1 after excluding Elixhauser comorbidity index from the list of model covariates.

	Model 1	Model 2	Model 3	Model 4	Model 5	Model 6
Outcome	Time from first encounter with provider to first AMG	Receipt of AMG at first visit with provider	Time from first diagnosis with dry eye to first AMG	Receipt of AMG at first diagnosis with dry eye	Time from first AMG to second AMG	Receipt of multiple AMG
Model specification	Cox proportional hazards	Logistic	Cox proportional hazards	Logistic	Cox proportional hazards	Logistic
Model N	N=23,059	N=21,220	N=6,547	N=8,538	N=9,012	N=23,066
Mean days to outcome	3,171		1,119		106	
Frequency of outcome		13		2,305		10,815
	HR (p-value)	OR (p-value)	HR (p-value)	OR (p-value)	HR (p-value)	OR (p-value)
Provider specialty						
Ophthalmology (base)						
Optometry	1.159 (p<0.001)	4.001 (p=0.029)	1.035 (p=0.171)	1.316 (p<0.001)	1.166 (p<0.001)	1.240 (p<0.001)
Other	1.465 (p<0.001)		1.379 (p=0.521)	3.229 (p=0.160)	1.564 (p=0.139)	1.879 (p=0.002)
Diagnosis associated with AMG						
Corneal abrasion (base)						
Corneal ulcer	1.120 (p<0.001)	0.633 (p=0.713)			1.100 (p=0.029)	1.329 (p<0.001)
Dry eye	0.908 (p<0.001)	0.896 (p=0.896)			1.486 (p<0.001)	2.427 (p<0.001)
Keratitis or conjunctivitis	0.846 (p<0.001)				1.183 (p=0.001)	1.716 (p<0.001)
Other	1.087 (p<0.001)	1.754 (p=0.506)			1.053 (p=0.205)	1.299 (p<0.001)
Sex						
Female (base)						
Male	0.987 (p=0.367)	1.322 (p=0.626)	1.285 (p<0.001)	1.770 (p<0.001)	1.044 (p=0.063)	0.886 (p<0.001)
Age (years)	0.998 (p=0.004)	0.986 (p=0.587)	0.976 (p<0.001)	0.974 (p<0.001)	1.000 (p=0.285)	0.995 (p<0.001)

Notes: HR=Hazard Ratio. OR=Odds Ratio.
