# Supplementary material for: Utilization Patterns and Costs of Ocular Amniotic Membrane Grafts in the Medicare Population
Source: Ophthalmology. Author manuscript; Available in PMC 2026 Jul 21. (PMC13387592; doi:10.1016/j.ophtha.2025.08.023)

Figure S3: Total claims for sutureless amniotic membrane grafts submitted by different ocular subspecialists in a 20% sample of Medicare Part B, 2011-2020

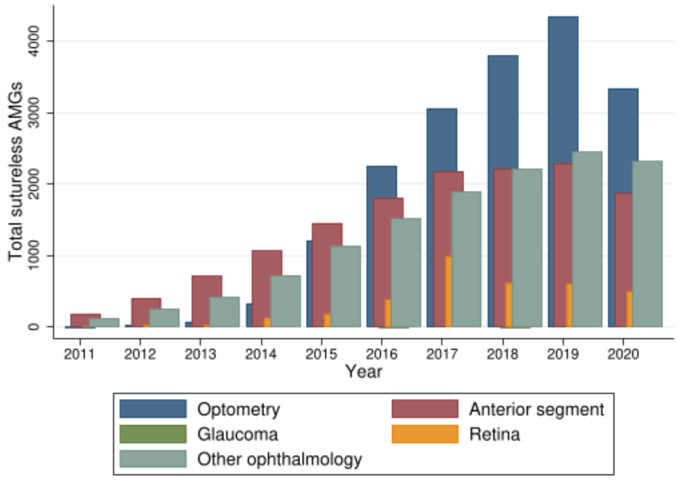

Supplement: 4 [file NIHMS2182128-supplement-4.pdf]
