# Supplementary material for: Utilization Patterns and Costs of Ocular Amniotic Membrane Grafts in the Medicare Population
Source: Ophthalmology. Author manuscript; Available in PMC 2026 Jul 21. (PMC13387592; doi:10.1016/j.ophtha.2025.08.023)
Supplement: 5 [file NIHMS2182128-supplement-5.pdf]

Figure S4: Primary diagnosis code associated with claims for sutureless amniotic membrane grafts from ophthalmologists and optometrists in a 20% sample of Medicare Part B, 2011-2020

A: Optometrists

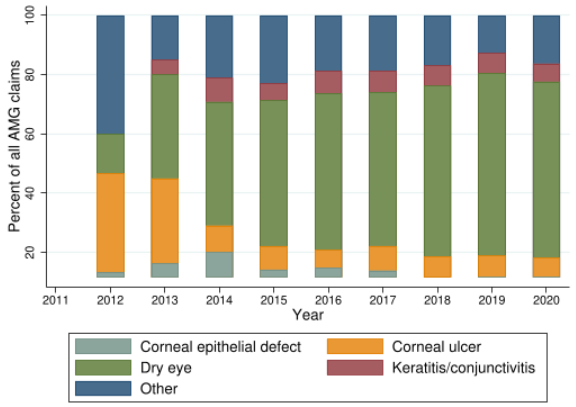

B: Ophthalmologists

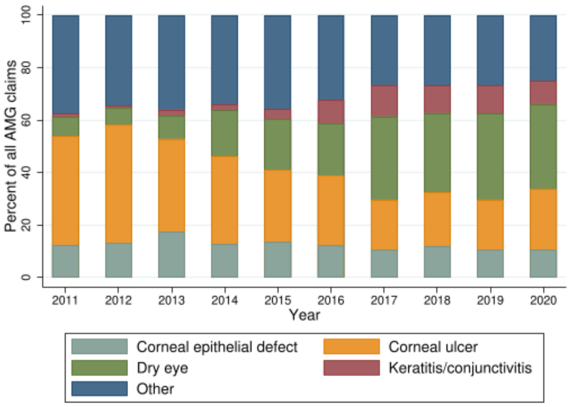

Appendix Figure 3: Average annual charges for a dry eye patient among ophthalmologists and optometrists in a 20% sample of Medicare Part B, 2011-2020

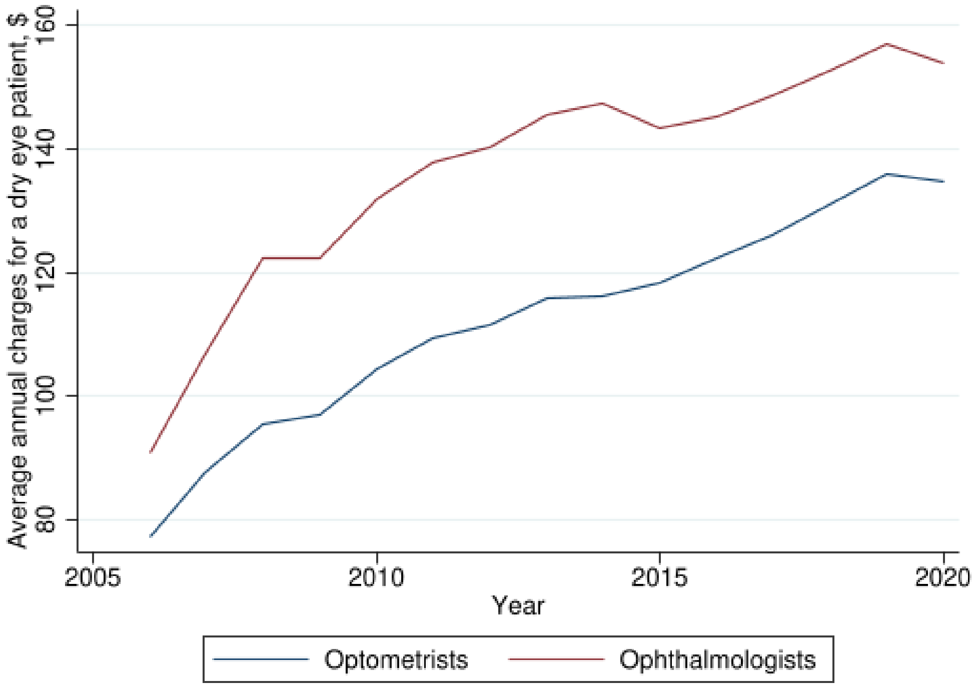

Average annual charges for a dry eye patient, $
160
140
120
100
80
2005
2010
2015
2020
Year
Optometrists
Ophthalmologists
