# Supplementary material for: Utilization Patterns and Costs of Ocular Amniotic Membrane Grafts in the Medicare Population
Source: Ophthalmology. Author manuscript; Available in PMC 2026 Jul 21. (PMC13387592; doi:10.1016/j.ophtha.2025.08.023)
Supplement: 1 [file NIHMS2182128-supplement-1.pdf]

Appendix Table 1

ICD9/ICD10 codes by diagnosis.																			
Elixhauser comorbidity index																			
Congestive heart failure	Cardiac arrhythmia	Valvular disease	Pulmonary circulation disorders	Peripheral vascular disorders	Hypertension, uncomplicated	Hypertension, complicated	Paralysis	Other neurological disorders	Chronic pulmonary disease	Diabetes, uncomplicated	Diabetes, complicated	Hypothyroidism	Renal failure	Liver disease	Peptic ulcer disease, excluding bleeding	AIDS/HIV	Lymphoma	Metastatic cancer	Solid tumor without metastasis
39891	99601	9320	I2601	930	4010	40200	G041	E7500	J40	E0800	E0821	E000	N183	B180	K254	B20	C8100	C770	C000
40201	99604	9321	I2602	4373	4011	40201	G800	E7501	J410	E0801	E0822	E001	N184	B181	K255	42	C8101	C771	C001
40211	4260	9322	I2609	4400	4019	40210	G801	E7502	J411	E0810	E0829	E002	N185	B182	K256		C8102	C772	C002
40291	42610	9323	I2690	4401	I10	40211	G802	E7509	J418	E0811	E08311	E009	N186	I8500	K257		C8103	C773	C003
40401	42611	9324	I2692	44020		40290	G804	E7510	J42	E089	E08319	E018	N189	I8501	K259		C8104	C774	C004
40403	42612	3940	I2699	44021		40291	G808	E7511	J430	E0900	E08321	E02	N19	I8510	K264		C8105	C775	C005
40411	42613	3941	I270	44022		40300	G809	E7519	J431	E0901	E08329	E030	Z4901	I8511	K265		C8106	C778	C006
40413	4267	3942	I271	44023		40301	G8100	E7523	J432	E0910	E08331	E031	Z4902	K700	K266		C8107	C779	C008
40491	4269	3949	I2720	44024		40310	G8101	E7525	J438	E0911	E08339	E032	Z9115	K702	K267		C8108	C7800	C009
40493	4270	3950	I2721	44029		40311	G8102	E7529	J439	E099	E08341	E033	Z940	K7030	K269		C8109	C7801	C01
4254	4271	3951	I2722	44030		40390	G8103	E754	J440	E1010	E08349	E038	Z992	K7031	K274		C8110	C7802	C020
4255	4272	3952	I2723	44031		40391	G8104	F842	J441	E1011	E08351	E039	Z4931	K7040	K275		C8111	C781	C021
4257	42731	3959	I2724	44032		40400	G8110	G10	J449	E109	E08359	E890	Z4932	K7041	K276		C8112	C782	C022
4258	42732	3960	I2729	4404		40401	G8111	G110	J4520	E1100	E0836	243	40301	K709	K277		C8113	C7830	C023
4259	42741	3961	I2781	4408		40402	G8112	G111	J4521	E1101	E0839	2440	40311	K7210	K279		C8114	C7839	C024
4280	42742	3962	I2782	4409		40403	G8113	G112	J4522	E119	E0840	2441	40391	K7211	K284		C8115	C784	C028
4281	42760	3963	I2783	44100		40410	G8114	G113	J4530	E1300	E0841	2442	40402	K7290	K285		C8116	C785	C029
42820	42761	3968	I2789	44101		40411	G8190	G114	J4531	E1301	E0842	2443	40403	K7291	K286		C8117	C786	C030
42821	42769	3969	I279	44102		40412	G8191	G118	J4532	E1310	E0843	2448	40412	K730	K287		C8118	C787	C031
42822	42781	3970	I280	44103		40413	G8192	G119	J4540	E1311	E0844	2449	40413	K731	K289		C8119	C7880	C039
42823	42789	3971	I288	4411		40490	G8193	G120	J4541	E139	E0849	2409	40492	K732	53170		C8120	C7889	C040
42830	4279	3979	I289	4412		40491	G8194	G121	J4542	O24011	E0851	2461	40493	K738	53171		C8121	C7900	C041
42831	7850	4240	4150	4413		40492	G8220	G1220	J4550	O24012	E0852	2468	5851	K739	53190		C8122	C7901	C048
42832	V4500	4241	41511	4414		40493	G8221	G1221	J4551	O24013	E0859		5852	K740	53191		C8123	C7902	C049
42833	V4501	4242	41512	4415		40501	G8222	G1222	J4552	O24019	E08610		5853	K741	53270		C8124	C7910	C050
42840	V4502	4243	41513	4416		40509	G8250	G1229	J45901	O2402	E08618		5854	K742	53271		C8125	C7911	C051
42841	V4509	42490	41519	4417		40511	G8251	G128	J45902	O2403	E08620		5855	K743	53290		C8126	C7919	C052
42842	V5331	42491	4160	4419		40519	G8252	G129	J45909	O24111	E08621		5856	K744	53291		C8127	C792	C058
42843	V5332	42499	4161	4431		40591	G8253	G132	J45990	O24112	E08622		5859	K745	53370		C8128	C7931	C059
4289	V5339	7463	4162	44321		40599	G8254	G138	J45991	O24113	E08628		586	K7460	53371		C8129	C7932	C060
I0981	I441	7464	4168	44322		I110	G830	G20	J45998	O24119	E08630		5880	K7469	53390		C8130	C7940	C061
I501	I442	7465	4169	44323		I119	G8310	G214	J470	O2412	E08638		V560	K754	53391		C8131	C7949	C062
I5020	I4430	7466	4170	44324		I120	G8311	G2401	J471	O2413	E08641		V561	K7581	53470		C8132	C7951	C0680
I5021	I4439	V422	4178	44329		I129	G8312	G2402	J479	O24311	E08649		V562	K760	53471		C8133	C7952	C0689
I5022	I456	V433	4179	44381		I130	G8313	G2409	J60	O24312	E0865		V5631	K766	53490		C8134	C7960	C069
I5023	I459	A5203		44382		I1310	G8314	G242	J61	O24313	E0869		V5632	K7689	53491		C8135	C7961	C07
I5030	I470	I050		44389		I1311	G8320	G248	J620	O24319	E088		V568	K769			C8136	C7962	C080
I5031	I471	I051		4439		I132	G8321	G254	J628	O2432	E0921		V4511	Z944			C8137	C7970	C081
I5032	I472	I052		4471		I150	G8322	G255	J630	O2433	E0922		V4512	7022			C8138	C7971	C089
I5033	I479	I058		5571		I151	G8323	G2581	J631	O24811	E0929		V420	7023			C8139	C7972	C090
I5040	I480	I059		5579		I152	G8324	G300	J632	O24812	E09311			7032			C8140	C7981	C091
I5041	I481	I060		V434		I158	G8330	G301	J633	O24813	E09319			7033			C8141	C7982	C098
I5042	I482	I061		I700		I159	G8331	G308	J634	O24819	E09321			7044			C8142	C7989	C099
I5043	I483	I062		I701			G8332	G309	J635	O2482	E09329			7054			C8143	C799	C100
I509	I484	I068		I70201			G8333	G3101	J636	O2483	E09331			4560			C8144	C7B00	C101
	I4891	I069		I70202			G8334	G3109	J64	O24911	E09339			4561			C8145	C7B01	C102
	I4892	I070		I70203			G834	G311	J65	O24912	E09341			45620			C8146	C7B02	C103
	I4901	I071		I70208			G835	G312	J660	O24913	E09349			45621			C8147	C7B03	C104
	I4902	I072		I70209			G8381	G3181	J661	O24919	E09351			570			C8148	C7B04	C108
	I491	I078		I70211			G8382	G3182	J662	O2492	E09359			5710			C8149	C7B09	C109
	I492	I079		I70212			G8383	G3183	J668	O2493	E0936			5711			C8170	C7B1	C110
	I493	I080		I70213			G8384	G3184	J670	25000	E0939			5712			C8171	C7B8	C111
	I4940	I081		I70218			G8389	G3185	J671	25001	E0940			5713			C8172	C800	C112
	I4949	I082		I70219			G839	G3189	J672	25002	E0941			57140			C8173	C801	C113
	I495	I083		I70221			I69031	G319	J673	25003	E0942			57141			C8174	R180	C118
	I498	I088		I70222			I69032	G3281	J674	25010	E0943			57142			C8175	1960	C119
	I499	I089		I70223			I69033	G35	J675	25011	E0944			57149			C8176	1961	C12
[illegible]																			

											Ophthalmology diagnoses							
Rheumatoid arthritis/collagen	Coagulopathy	Obesity	Weight loss	Fluid and electrolyte disorders	Blood loss anemia	Deficiency anemia	Alcohol abuse	Drug abuse	Psychoses	Depression	Dry eye	Corneal ulcer	Neurotrophic corneal ulcer	Recurrent corneal erosion	Corneal abrasion	Keratoconjunctivitis	Corneal edema	Eyelid abnormalities
L900	D65	E6601	E40	E860	D500	D501	F1010	F1110	F200	F320	H16221	H16001	H16231	H18831	S0500XA	H168	H1810	H02001
L940	D66	E6609	E41	E861	O9081	D508	F10120	F11120	F201	F321	H16222	H16002	H16232	H18832	S0500XD	H169	H1811	H02002
L941	D67	E661	E42	E869	O99011	D509	F10121	F11121	F202	F322	H16223	H16003	H16233	H18833	S0500XS	H16241	H1812	H02003
L943	D680	E662	E43	E870	O99012	D510	F10129	F11122	F203	F323	H16229	H16009	H16239	H18839	S0501XA	H16242	H1813	H02004
M0500	D681	E668	E440	E871	O99013	D511	F1014	F11129	F205	F328	H04121	H16011	37035	37142	S0501XD	H16243	H1820	H02005
M05011	D682	E669	E441	E872	O99019	D512	F10150	F1114	F2081	F3281	H04122	H16012			S0501XS	H16249	H18211	H02006
M05012	D68311	O99210	E45	E873	O9902	D513	F10151	F11150	F2089	F3289	H04123	H16013			S0502XA	H16251	H18212	H02009
M05019	D68312	O99211	E46	E874	O9903	D518	F10159	F11151	F209	F329	H04129	H16019			S0502XD	H16252	H18213	H02011
M05021	D68318	O99212	E640	E875	2800	D519	F10180	F11159	F22	F330	H16141	H16021			S0502XS	H16253	H18219	H02012
M05022	D6832	O99213	R634	E876		D520	F10181	F11181	F23	F331	H16142	H16022			9181	H16259	H18221	H02013
M05029	D684	O99214	R636	E8770		D521	F10182	F11182	F24	F332	H16143	H16023			9182	H16261	H18222	H02014
M05031	D688	O99215	260	E8771		D528	F10188	F11188	F250	F333	H16149	H16029			9189	H16262	H18223	H02015
M05032	D689	R939	261	E8779		D529	F1019	F1119	F251	F338	H16201	H16031				H16263	H18229	H02016
M05039	D691	Z6830	262	E878		D530	F1020	F1120	F258	F339	H16202	H16032				H16269	H18231	H02019
M05041	D693	Z6831	2630	2760		D531	F1021	F1121	F259	F341	H16203	H16033				H16291	H18232	H02021
M05042	D6941	Z6832	2631	2761		D532	F10220	F11220	F28	F4321	H16209	H16039				H16292	H18233	H02022
M05049	D6942	Z6833	2632	2762		D538	F10221	F11221	F29	29620	H16211	H16041				H16293	H18239	H02023
M05051	D6949	Z6834	2638	2763		D539	F10229	F11222	F3010	29621	H16212	H16042				H16299	H1830	H02024
M05052	D6951	Z6835	2639	2764		D630	F10230	F11229	F3011	29622	H16213	H16043				37022	H18311	H02025
M05059	D6959	Z6836	78321	27650		D631	F10231	F1123	F3012	29623	H16219	H16049				37023	H18312	H02026
M05061	D696	Z6837	78322	27651		D638	F10232	F1124	F3013	29624	37020	H16051				37024	H18313	H02029
M05062	D7582	Z6838	7994	27652		D649	F10239	F11250	F302	29625	37021	H16052				37031	H18319	H02031
M05069	O99111	Z6839		27661		2801	F1024	F11251	F303	29626	37033	H16053				37032	H18321	H02032
M05071	O99112	Z6841		27669		2808	F10250	F11259	F304	29630	37034	H16059				37040	H18322	H02033
M05072	O99113	Z6842		2767		2809	F10251	F11281	F308	29631	37040	H16061				37044	H18323	H02034
M05079	O99119	Z6843		2768		2810	F10259	F11282	F309	29632	37513	H16062				37049	H18329	H02035
M0509	O9912	Z6844		2769		2811	F1026	F11288	F310	29633	37514	H16063				H10011	H18331	H02036
M0510	O9913	Z6845		2536		2812	F1027	F1129	F3110	29634	37515	H16069				H10012	H18332	H02039
M05111	2860	Z6854				2813	F10280	F1210	F3111	29635	7102	H16071				H10013	H18333	H02041
M05112	2861	27800				2814	F10281	F12120	F3112	29636		H16072				H10019	H18339	H02042
M05119	2862	27801				2818	F10282	F12121	F3113	29650		H16073				H10021		H02043
M05121	2863	27802				2819	F10288	F12122	F312	29651		H16079				H10022		H02044
M05122	2864	27803					F1029	F12129	F3130	29652		37000				H10023		H02045
M05129	28652						F10921	F12150	F3131	29653		37001				H10029		H02046
M05131	28653						F1094	F12151	F3132	29654		37002				H1010		H02049
M05132	28659						F10950	F12159	F314	29655		37003				H1011		H02051
M05139	2866						F10951	F12180	F315	29656		37004				H1012		H02052
M05141	2867						F10959	F12188	F3160	3004		37005				H1013		H02053
M05142	2869						F1096	F1219	F3161	3090		37006				H10211		H02054
M05149	2871						F1097	F1220	F3162	3091		37007				H10212		H02055
M05151	28730						F10980	F1221	F3163	30921						H10213		H02056
M05152	28731						F10981	F12220	F3164	30922						H10219		H02059
M05159	28732						F10982	F12221	F3170	30923						H10221		H02101
M05161	28733						F10988	F12222	F3171	30924						H10222		H02102
M05162	28739						F1099	F12229	F3172	30928						H10223		H02103
M05169	28741						2652	F12250	F3173	30929						H10229		H02104
M05171	28749						2911	F12251	F3174	3093						H10231		H02105
M05172	2875						2912	F12259	F3175	3094						H10232		H02106
M05179							2913	F12280	F3176	30981						H10233		H02109
M0519							2915	F12288	F3177	30982						H10239		H02111
M0520							29181	F1229	F3178	30983						H1030		H02112
M05211							29182	F1310	F3181	30989						H1031		H02113
M05212							29189	F13120	F3189	3099						H1032		H02114
M05219							2919	F13121	F319	311						H1033		H02115
M05221							30300	F13129	F324							H10401		H02116
M05222							30301	F1314	F325							H10402		H02119
M05229							30302	F13150	F3340							H10403		H02121
[illegible]																		
